# Potent suppression of HIV viral replication by a novel inhibitor of Tat

**DOI:** 10.1186/1742-4690-9-S1-O11

**Published:** 2012-05-25

**Authors:** Guillaume Mousseau, Mark A Clementz, Wendy N Bakeman, Nisha Nagarsheth, Michael Cameron, Jun Shi, Phil Baran, Rémi Fromentin, Nicolas Chomont, Susana T Valente

**Affiliations:** 1 Department of Infectology, The Scripps Research Institute, Jupiter, Florida, USA; 2 Department of Molecular Therapeutics and Translational Research Institute, The Scripps Research Institute, Jupiter, Florida, USA; 3 Department of Chemistry, The Scripps Research Institute, La Jolla, California, USA; 4 Vaccine and Gene Therapy Institute, Port St. Lucie, Florida, USA

## Background

Tat, the HIV Trans-Activator of Transcription is a potential antiviral target. Tat binds to the 5’ terminal region of HIV mRNAs stem-bulge-loop structure called the Trans-activation Responsive (TAR) element and activates transcription from the HIV promoter. Plasma viremia stubbornly persists in HIV-1 infected subjects despite receiving HAART, suggesting that residual levels of viral production originate from an integrated form of the HIV genome that is continuously transcribed at low levels. As current antiretrovirals (ARVs) fail to inhibit transcription from integrated viral genomes or viral production from stable cellular reservoirs, novel classes of ARVs are needed to inhibit this process.

## Results

Cortistatin A is a steroidal alkaloid isolated from the marine sponge *Corticium simplex*. Here, we show that its analog didehydro-Cortistatin A (dCA) inhibits Tat-mediated trans-activation of the integrated HIV provirus by binding specifically to the TAR-binding domain of Tat. dCA reduces cell-associated viral RNA and capsid p24 antigen production in acutely and chronically infected cultured and primary cells, at a half maximal effective concentration (EC_50_) of 0.7 pM to 2.5 nM, depending on the multiplicity of infection (MOI). dCA reduces both transcriptional initiation/elongation from the viral promoter and alters the nucleolar localization of Tat. Termination of dCA treatment does not result in immediate virus rebound as the HIV promoter is transcriptionally silenced. dCA inhibits both HIV-1 and HIV-2, and displays high bioavailability. dCA added to a combination of ARVs mediates a statistically significant reduction in viral replication from primary CD4^+^T cells isolated from viremic patients compared to the ARVs alone, and abrogates low-level virus replication from CD4^+^T cells isolated from aviremic patients undergoing HAART treatment.

## Conclusions

With a therapeutic index of over 8000, dCA defines a novel class of HIV anti-viral drugs endowed with the ability to decrease residual viremia during HAART, and should be considered as a promising drug to be included in therapeutic eradication strategies.

